# Development of a Nurse Turnover Prediction Model in Korea Using Machine Learning

**DOI:** 10.3390/healthcare11111583

**Published:** 2023-05-28

**Authors:** Seong-Kwang Kim, Eun-Joo Kim, Hye-Kyeong Kim, Sung-Sook Song, Bit-Na Park, Kyoung-Won Jo

**Affiliations:** 1 Department of Nursing, Gangneung-Wonju National University, Wonju City 20403, Republic of Koreajowon2006@naver.com (K.-W.J.); 2 Department of Nursing, Inha University, Incheon 22212, Republic of Korea

**Keywords:** machine learning, random forest, nurses, turnover, prediction

## Abstract

Nurse turnover is a critical issue in Korea, as it affects the quality of patient care and increases the financial burden on healthcare systems. To address this problem, this study aimed to develop and evaluate a machine learning-based prediction model for nurse turnover in Korea and analyze factors influencing nurse turnover. The study was conducted in two phases: building the prediction model and evaluating its performance. Three models, namely, decision tree, logistic regression, and random forest were evaluated and compared to build the nurse turnover prediction model. The importance of turnover decision factors was also analyzed. The random forest model showed the highest accuracy of 0.97. The accuracy of turnover prediction within one year was improved to 98.9% with the optimized random forest. Salary was the most important decision factor for nurse turnover. The nurse turnover prediction model developed in this study can efficiently predict nurse turnover in Korea with minimal personnel and cost through machine learning. The model can effectively manage nurse turnover in a cost-effective manner if utilized in hospitals or nursing units.

## 1. Introduction

There were 436,340 nurses in South Korea as of 2020, representing an 11.4% increase from 391,493 in 2019 and an annual growth rate of approximately 6.2% [1]. However, the number of nurses working in hospitals is only half of the OECD average (As of 2020, the OECD average number of clinical nurses per 1000 population is 7.6, while in South Korea, the number is 3.8). More than 86.2% of hospital-level medical institutions do not meet the nursing personnel standards [1,2]. The average turnover rate for Korean nurses in hospitals has steadily increased from approximately 11.6% in 2019 to about 16% in 2021 for major general hospitals and has continued to increase annually [3]. However, this is not only a problem unique to South Korea. Globally, the turnover rate for nurses is generally high, ranging from 15% to 43%, and this has been reported to have a negative impact on the medical sector [4]. Increased nurse turnover can lead to job stress, nursing errors, increased patient mortality rates, increased workload for remaining nurses, reduced organizational commitment, and a decline in overall nursing service quality [5]. Therefore, various studies have been conducted to predict nurse turnover and understand nurses’ intentions to leave their jobs.

Turnover is defined as a voluntary termination of a member’s role in an organization, with the member receiving monetary compensation and moving from within to outside the organization [6]. Turnover intention is defined as the perceived likelihood of an individual permanently leaving an organization in the near future [7]. A systematic literature review of the factors influencing turnover intention among South Korean nurses revealed that various and complex factors, including demographic characteristics such as age, clinical experience, and marital status, as well as job satisfaction, job stress, organizational commitment, and ward organizational culture, influence the decision to leave [2]. However, in cases where actual turnover occurred, salary, age, the distance between home and company, length of service, and other factors were the determining factors in the decision to leave [8]. Therefore, there may be differences between the factors influencing turnover intention and the actual turnover decision.

The term “employee turnover” refers to the voluntary separation (in which employees receive monetary compensation for moving outside of the organization) or involuntary separation (which is determined by the organization’s business conditions) of individuals from an organization [9]. Additionally, turnover can be classified as avoidable, which is influenced by factors such as working conditions, wages, and benefits that are controllable by the organization, and unavoidable, which is influenced by factors such as personal health, family circumstances, and individual situations that are beyond the control of the organization [9]. Young people tend to experience more significant changes in their life cycle (e.g., independence, marriage, childbirth, and child-rearing) more than middle-aged people. Thus, they often seek to find a suitable job through multiple turnovers rather than finding their desired job all at once [10]. Therefore, turnover usually occurs before employees have gained proficiency in their job, and it often falls under the categories of voluntary and unavoidable turnover [10]. Thus, efforts to predict employee turnover should focus on the reasons for involuntary and avoidable turnover, as these are areas that organizations can control.

Various domestic and international studies have been conducted to predict employee turnover and understand the reasons for turnover. In a study on employee turnover prediction using artificial intelligence (AI) at a Chinese telecommunications company, salary was the most important factor in the decision to leave, followed by overtime hours, age, distance between home and work, and length of employment [8]. For Korean nurses, demographic factors such as age, sex, marital status, work experience, and job position were found to be more common reasons for turnover compared with complex factors [2,11,12]. According to a 2022 study by the Health Insurance Review & Assessment Service, turnover rates for nurses were higher for those under 30 years, those with less than one year of clinical experience, and male nurses. Organizational factors such as working in a special department, being a non-regular employee, and having a lower nursing grade were also found to be associated with higher turnover rates [11]. In Korea, attempts have also been made to predict turnover rates for new nurses using AI [13]. Similar to previous studies, personal and organizational factors were used to predict turnover, and the most frequent deciding factors for turnover were found to be marriage, childbirth, and child-rearing [13]. If the turnover decision factors revealed through previous studies can be utilized to predict turnover rates and understand the reasons for turnover among nurses, it is expected that proactive hiring plans can be made and measures can be taken to reduce the possibility of resignations.

To predict turnover, it is necessary to learn and infer from a vast amount of relevant data, as there are various factors associated with turnover. Artificial intelligence (AI) makes this possible. Among the concepts of AI, machine learning aims to uncover hidden insights and complex patterns in tasks such as text analysis or prediction model construction [14]. Machine learning effectively extracts regularities and makes repeatable decisions in tasks related to high-dimensional data, such as classification, regression, and clustering, based on the given problem and available data through supervised or unsupervised learning. Thus, machine learning is widely used as a predictive technology in many fields [14].

Using predictive technology, it is anticipated that effective hospital and ward management will be possible if controllable nurse turnover can be predicted and proactively addressed. 

This study aimed to develop and evaluate a predictive model for nurses’ turnover in Korea using machine learning. Specifically, the objectives are as follows: (1) to collect and preprocess data for model construction, including data classification and assignment for training; (2) to select and evaluate an effective nurse turnover prediction model; and (3) to identify and analyze the importance of variables that affect the nurse turnover prediction model.

## 2. Materials and Methods

### 2.1. Design and Subjects

This study was a secondary data analysis research. The study was conducted in two stages: construction and evaluation of the prediction model. The subjects were nurses who worked or had retired from a tertiary hospital in * city from 1 January 2010 to 31 July 2021.

### 2.2. Construction of the Prediction Model

#### 2.2.1. Data Collection

Training and testing data were needed to generate the model. Sociodemographic and job-related data from 630 retired and 780 employed nurses in the nursing department of the general hospital were collected from 1 January 2011 to 31 July 2021. Sociodemographic characteristics included age, sex, residential area, use of dormitory, and marital status. Job-related characteristics included department, year of employment, year of resignation, salary, and length of employment. The sociodemographic and job-related data of the resigned and employed nurses were obtained as coded information from the hospital’s personnel department.

#### 2.2.2. Structure of the Nurse Turnover Prediction Model

**Figure 1:**
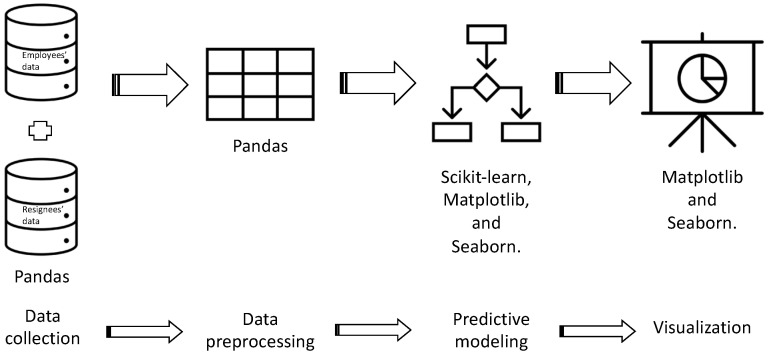
Structuring Process of the Nurse Turnover Predication Model.

The data necessary for building the nurses’ turnover prediction model were modified. To utilize well-constructed library sources in this process, an open-source structure in Python programming language was used. The libraries included for constructing this structure were Pandas, Scikit-learn, Matplotlib, and Seaborn. Briefly, Pandas “https://pandas.pydata.org/ (accessed on 10 August 2021) was used for data preprocessing and feature engineering, and Scikit-learn “https://scikit-learn.org/stable/ (accessed on 10 August 2021)” was used for machine learning. Meanwhile, Matplotlib “https://matplotlib.org/ (accessed on 10 August 2021)” and Seaborn “https://seaborn.pydata.org/ (accessed on 10 August 2021)” were used to visualize the results of the prediction model in tables and graphs (Figure 1). 

#### 2.2.3. Data Preprocessing

The following preprocessing steps were performed to convert the data into the format required for the error matrix of the prediction model:

1. Natural language, sentence punctuation, and numbers were converted into vectors (numbers) that the computer can understand. This was done using techniques such as binary, rank, or categorical encoding.

Natural language is mainly text data, which must be converted into numerical data that the computer can understand. To do this, we used natural language processing technology.

We also removed punctuation from the sentences because they are treated as unnecessary elements in the text.

Numerical data plays an important role in data analysis. Therefore, this data was converted into a form the computer can understand.

2. Vectorized data were normalized and standardized to achieve high performance.

Normalization scales the data to distribute all values within a certain range.

Standardization involves converting a distribution into a normal distribution by matching the mean of the data to zero and the variance to one.

3. Each row was indexed, and the variables were treated as nominal variables with 1 and 0. For example, sex was dummy-coded as 1 (female) and 0 (male). Residential areas were treated as ranking variables in the order of their proximity to the institution to which the researcher belongs.

The index separates data by giving each row a unique number.

A nominal variable is a variable that represents one of several distinct categories. Sex is treated as a nominal variable because it has only two values: male and female.

A rank variable is a variable that represents one of several distinct categories. Residential areas are treated as ranking variables by assigning values from 1 to 13 in order of closeness to the institution. Variables such as Team and Grade were treated as categories. For example, Registered Nurses were coded as 1, Charge Nurses as 2, and Nurse Practitioners as 3.

4. The resignation date was classified as the number of years of service from 1 year to 24 years, and the resignation status is set to Y value for each year.

The number of years of service was calculated using the employee’s start date and departure date.

The resignation date was used to predict nurses’ decision to quit.

5. Null or outlier values in the collected data were removed and not used in the model. This was done to ensure the quality of the data used for training and testing the model.

#### 2.2.4. Construction of Training Data, Validation Data, and Data Set

After preprocessing the collected data, the data to be analyzed were divided into training datasets and validation datasets. The target data and feature data were separated, with the target data being the column containing retirement information of retired nurses and the feature data being the variables that explain the target data, excluding the column containing retirement information. 

#### 2.2.5. Proposal for the Predictive Model

##### Decision Tree

The decision tree is a method of predicting patterns in data by representing them as a combination of predictable rules, which resemble the shape of a tree [15]. Decision trees are capable of classification and regression and can predict categorical and continuous variables. They are an analytical method for classifying or predicting the target group by charting decision rules [16]. Owing to the tree structure, decision trees are more easily understood and explained by researchers compared with other methods such as discriminant analysis, regression analysis, or neural networks [15]. Although decision trees can be used in any case of classification or prediction, they are more useful when an explanation of the analytical process is required rather than the accuracy of the analysis. Therefore, decision trees are often used to find rules from data and predict future events based on them [14]. In this study, we have used input variables (X), such as sex, age, marital status, and dormitory status, to determine the splitting criteria and branching in tree form. 

##### Logistic Regression

Logistic regression is like linear regression, but it can be considered a classification technique because the result of the given input data is divided into a specific category [15]. Unlike linear regression, logistic regression predicts the probability of the data belonging to a certain category as a binary value between 0 and 1, using regression. Therefore, machine learning requires supervised learning using values. In this study, 80% of the overall data were assigned as training data, and 20% were assigned as validation data to assess the performance of the classification.

##### Random Forest

Random forest is an algorithm that predicts the dependent variable (Y) by generating multiple decision trees, taking the majority vote or the average of the results, and using bootstrapping to extract various samples during the learning process [8,16]. In this study, we used variables excluding retire as input variables (X) and secured diversity in decision trees through data sampling and variable (X) selection. Random forest can rank the importance of attributes or variables in regression or classification problems [8,17], allowing us to identify X that significantly impacts Y in personnel analysis. Random forest consists of multiple decision trees [8,16]. In decision trees, we can determine how much an attribute can reduce impurity at a node and derive the importance of X by weighting the information gain ratio obtained through the weighted average [8]. Therefore, it reduces prediction variability, prevents overfitting, and has strengths in dealing with missing values.

### 2.3. Evaluation of the Prediction Model

We used a confusion matrix to calculate the classification report to evaluate whether binary classification models such as random forest were the best option [8]. The confusion matrix represents real-world situations where predictions are different from the ideal scenario where positives and negatives (or yes/no) are separated. In the current study, because the goal was to predict nurse turnover, we used precision (the ratio of true positives to all positive predictions), recall (the ratio of true positives to all actual positives), and accuracy (the ratio of correct predictions to total predictions) to evaluate the predictive ability of the model [8,16]. However, evaluating the model using only recall scores for turnover data can lead to low accuracy. Thus, we used the area under the curve (AUC) score to more accurately determine the predictive power for both target groups (current and former employees) [8,16]. The AUC score is a score that maximizes both recall and specificity (TN/FP + TN) scores, plotted on a graph where the vertical axis represents recall, and the horizontal axis represents 1-specificity. The receiver operating characteristic (ROC) curve is drawn with the X = Y line as the baseline, and the AUC score is calculated for the area above the baseline of the ROC curve. This approach allows the predictive model to accurately classify data using both recall and specificity. AUC represents the area under the ROC curve, and scores between 0.5 and 0.6 are inadequate, scores between 0.6 and 0.7 are typical, scores between 0.7 and 0.8 are good, scores between 0.8 and 0.9 are very good, and scores above 0.9 are considered excellent [16] (Table 1). 

## 3. Results

### 3.1. Sociodemographic Characteristics of the Participants

In the resigned group (*n* = 629), the majority were in their 20s (*n =* 305 participants, 48.5%) and were female (*n =* 577 participants, 91.7%). Further, most of the resignees belonged to a ward nursing team (*n =* 299 participants, 47.5%) and had a nursing position as a general nurse (*n =* 614 participants, 97.6%). The average salary was approximately KRW 41.9 million, and 70.3% (442 participants) used dormitories. In addition, 81.6% (513 participants) were married, and 86.2% (542 participants) were originally from Gangwon Province. In the currently employed group (*n =* 777), the majority were in their 20s (*n =* 475 participants, 61.1%) and were female (*n =* 718 participants, 92.4%). More than half of them (*n =* 399 participants, 51.4%) belonged to a ward nursing team, and the nursing position was the most common, at 91.6% (712 participants). The average salary was approximately KRW 51.1 million. Only 19.4% (150 participants) used dormitories, and 79.3% (616 participants) were unmarried. A total of 92% (*n =* 715) of the participants were originally from Gangwon Province. Table 2 provides more detailed information on the subjects’ sociodemographic characteristics.

### 3.2. Data Preprocessing and Learning Data Assignment

To optimize model performance, we performed data preprocessing on several nominal variables, including “sex”, “team”, “grade”, “dormitory”, “married”, and “Distance from home to workplace”. For “sex”, male and female sex were denoted as 0 and 1, respectively. “Team” represented the nurse’s department and was categorized as 1 for outpatient, 2 for intensive care unit, 3 for general ward, and 4 for nursing administration department. “Grade” reflected the nurse’s job title, with 1 denoting nurse, 2 denoting specialized nurse, and 3 denoting head nurse. “Dormitory” and “married” were replaced with 1 if applicable and 0 otherwise. “Age” and “income” were continuous variables, and “age” was determined as the difference between the current year and the year of birth. “Income” was preprocessed to include only whole numbers. Finally, “Distance from home to workplace” was classified only up to the province level, with a ranking assigned based on proximity to the researcher’s affiliated tertiary hospital. For example, K was assigned 1 and S was assigned 2. To predict nurse turnover, “resign” was designated as the Y variable, and the remaining variables were designated as X variables for the classification model. A total of 20% of the entire dataset were allocated to the test dataset.

### 3.3. Confusion Matrix of Nurse Turnover Prediction

**Figure 2:**
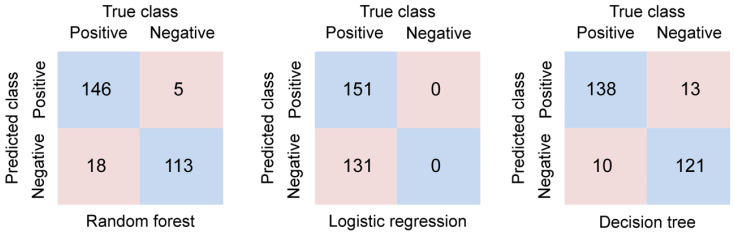
Confusion matrix of prediction models.

We constructed a confusion matrix using validation data to evaluate the predictive model for nurse turnover. A total of 20% of the entire dataset were allocated to the test dataset, with a sample size of 282. For the true positive (TP) area (i.e., cases predicted as resignees and those who actually resigned), logistic regression had the highest rate, predicting 151 out of 282 individuals. Meanwhile, the decision tree had the lowest prediction rate, predicting 138 out of 282 individuals. For the false negative (FN) area (i.e., cases predicted as non-resignees but who actually resigned), logistic regression also had the highest rate, predicting only 131 out of 282 individuals. The decision tree had the lowest classification performance, predicting only 10 out of 282 individuals. With respect to the false positive (FP) area (i.e., cases predicted as resignees but actually non-resignees), decision tree showed the highest rate, predicting 13 out of 282 individuals, while logistic regression showed the lowest, predicting 0 individuals. For the true negative (TN) area (i.e., cases predicted as non-resignees and actually non-resignees), the decision tree had the highest rate, predicting 121 out of 282 individuals. In contrast, logistic regression had the lowest rate, predicting 0 individuals (Figure 2).

### 3.4. Performance of Nurse Turnover Prediction Model

**Figure 3:**
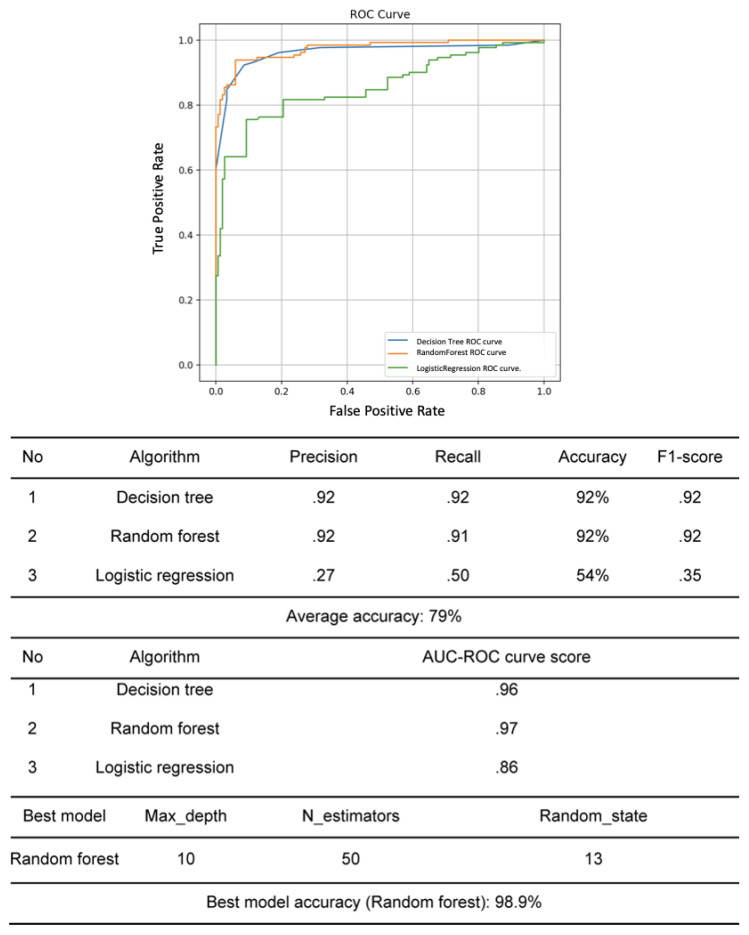
Comparison of prediction model performance.

First, random forest and decision tree showed similar precisions at 92%, while logistic regression had a significantly lower precision at 27%. Decision tree had the highest recall ability at 92%, while logistic regression had the lowest ability at 50%. Random forest and decision tree had similar accuracy at 92%, while logistic regression had a lower accuracy at 54%. The AUC-ROC curve score was the highest for random forest at 0.97. Therefore, the random forest model was identified as the most effective model for predicting turnover and was improved by implementing hyperparameter optimization. The accuracy of the improved random forest model in predicting turnover within one year was 98.9% (Figure 3).

### 3.5. Variable Importance in the Prediction Model

**Figure 4:**
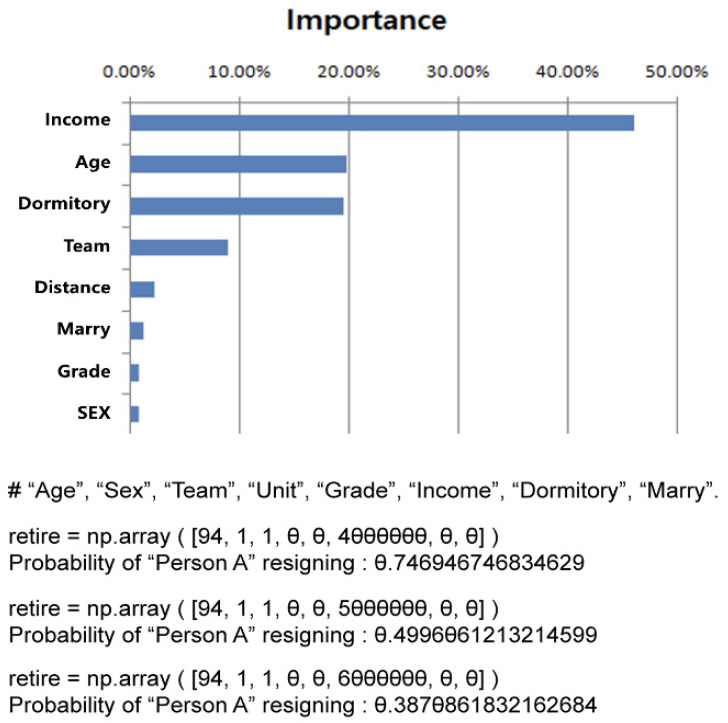
Random forest feature importance.

Among the variables in the random forest model, salary had the highest importance at 46.2%, followed by age at 19.8%. The importance of dormitory usage was 19.6%; department, 9%; place of residence, 2.3%; marital status, 1.3%; and rank and sex, both 0.9% (Figure 4). When all factors were held constant and only salary, which had the highest importance, was changed, the turnover rate for “person A” was the same as in Figure 4.

## 4. Discussion

Predicting nurse turnover entails learning and inferring from a vast amount of relevant data because various factors are associated with turnover. This study attempted to predict nurses’ turnover in Korea using machine learning. The turnover prediction model using the random forest algorithm demonstrated the highest predictive power, with an accuracy of 98.9%. 

The random forest algorithm has been used for decades and has been evaluated as a representative classification prediction model owing to its excellent algorithm and high predictive accuracy, as confirmed through numerous empirical studies [17]. Nurse turnover prediction models using random forest developed by Chakraborty et al. [18] and Ganthi et al. [19] showed prediction accuracies of 90% and 88%, respectively. In Korea, Choi et al. [13] used TensorFlow’s machine learning technology to predict nurse turnover. However, it could not provide the causes or solutions for predicted turnover based on the individual characteristics of the subjects, and its prediction rate was lower than that in the current study, at only 88.7% [13]. In addition to nurse turnover, turnover among telecommunications company employees [8] and customer service employees [15] has also been predicted using machine learning models. Therefore, if an accurate nurse turnover prediction model is utilized in nursing organizations, it is expected to enable effective human resource management and provide economic benefits, such as addressing problems in organizational management caused by sudden turnover and reducing costs associated with employing replacement personnel [11]. 

The most important variable in the prediction model was salary. In a study predicting job transitions among telecommunications company employees, salary was also the most influential factor in job decisions [8]. For Korean nurses, salary levels vary widely depending on clinical experience, position, type of healthcare facility, region, ward size, and other factors [20]. Furthermore, salary levels differ significantly even among nurses with the same clinical experience, which was identified as one of the direct factors affecting nurses’ turnover [20]. In Korea, nurses are unable to independently open their own practices due to medical regulations and must be employed by healthcare institutions. Therefore, healthcare institutions have a monopoly over the demand for nursing personnel, which leads to nurses receiving compensation that is less than what they contribute to production [21]. Salary is a representative external reward and a means of acknowledging the value of labor, in addition to promotion [22]. Imbalances between nurses’ efforts and rewards lead to burnout and work-related stress, which can result in job transitions [2]. Therefore, hospitals and nursing organizations require systemic support to increase nurses’ sense of achievement and provide fair compensation for their efforts to reduce job turnover rates. 

The second most important factor in the decision to change jobs was age. The high turnover rate among nurses in their 20s and 30s was similar to the results of studies conducted by the Hospital Nurse Association and the Health Insurance Review & Assessment Service [11,23]. Most previous studies identified age and short employment length as major factors in nurses’ turnover [2,11,12,13]. As of 2019, the average length of employment for nurses is seven years and seven months [23], which is less than half the average length of employment of 15 years and two months for all workers as of 2022, according to Statistics Korea [24]. The turnover of young nurses is thought to be due to insufficient education and training, a gap between theory and practice, unfamiliarity with the job, excessive workload, and stress [25]. Internal factors such as fear of responsibility in an unfamiliar environment, physical pain, and loss of confidence are also likely to play a role in turnover among young nurses [25]. Turnover due to age is considered a controllable factor within an organization, and it may be necessary for middle managers to intervene by providing sufficient education and training time, optimizing work assignments to minimize confusion between theory and practice, and only gradually increasing the workload to help new nurses adapt to the unfamiliar environment.

The dormitory was identified as the third most important factor in the decision to resign. Approximately 70% of resignees used the dormitory, while approximately 81% of current employees did not. Using the dormitory means workers live near their workplace, away from their families and familiar environment. Among nurses, the turnover rate is higher for those living alone than those living with their family, relatives, or dependent children [26]. In this study, the opposite trend was observed in the proportion of dormitory use between resignees and current employees, suggesting that those who use the dormitory may have decided to resign due to a lack of social support from nearby acquaintances and the challenge of living in an unfamiliar environment. However, there is still limited research on the use of dormitories or residency as a factor in nurses’ turnover decisions. Thus, these findings should be generalized cautiously, and further research on this topic is also needed.

The Department of Employment showed moderate importance as an influencing factor in turnover decisions. The results showed that ward nurses had the highest turnover rate, followed by the outpatient department; the nursing administration department had the lowest turnover rate. This could be because most of the study subjects were ward nurses, and therefore, the number of nurses who left the job was also the highest for these nurses. However, a previous study that predicted turnover among nurses in a single university hospital also showed similar results, with the highest turnover rate in the ward [13]. In contrast, a study conducted by the National Health Insurance Service on the hospital-based nurse grade or higher showed a high turnover rate in special departments such as the emergency room and intensive care unit [11], and contrasting results were observed in the current study. In the case of outpatient departments, some studies divide them into special departments; thus, it is necessary to be cautious in interpreting the results. Special departments have a high demand for skilled abilities, such as high work intensity, working within high-stress environments, operating the latest medical equipment, and monitoring and judging patient conditions. Occupational stress highly influences turnover [27]. In such cases, positive factors such as job satisfaction and organizational commitment can lower the intention to leave [2]. In addition, checking the personal tendencies and needs of nurses in detail and managing the workforce, such as department placement, is necessary to motivate work, improve nurse capabilities, and adapt the work department. As such, preventing turnover can be achieved by assigning a realistic workload to nurses with high work intensity or who are not yet skilled and by supplementing their shortcomings to boost their morale.

Sex was found to be the least important factor in the current analysis. Previous studies on nursing turnover have shown that female nurses have a higher turnover rate, but the results were not significant [11,12]. Meanwhile, one study found a higher turnover rate among male nurses [11]. In a study that predicted nursing turnover among university hospital nurses, sex was not identified as an influencing factor for turnover decision [13]. Therefore, it can be inferred that sex is not a major factor influencing turnover decisions. There was no significant difference in residence, marital status, and job position between the employees who did and did not stay in the current study. However, marital status has been reported as a significant factor in predicting nurses’ intention [2] and decisions [12,13] to leave. Thus, if there are employees whose marital status has changed, there is a need to focus on continuous attention and nursing management interventions to prevent attrition in the nursing workforce. Additionally, a negative atmosphere during the decision-making process was reported to make nurses considering leaving feel unsupported by their supervisors and colleagues and may cause them to conceal their thoughts about leaving [25]. As such, efforts need to be made to freely discuss reasons for leaving and continuously improve the underlying problems.

The advantages of this study were as follows: First, a machine learning model was used to effectively predict nurses’ turnover rates in Korea, and the model showed high prediction rates and provided a basis for further academic research. Second, by identifying and analyzing important variables that affect nurses’ turnover, this study can contribute to the efficient management of the nursing workforce.

However, it is important to note that this study has several limitations. Since the research was conducted on nursing personnel in a single tertiary hospital, the generalizability of the findings may be limited. Including additional personal and organizational factors, such as nurses’ personalities, experiences, organizational culture, and leadership, may improve the model’s predictive ability and provide a more comprehensive understanding of nursing turnover.

Furthermore, this study did not account for external factors such as economic conditions or policy changes that could influence nursing turnover. Future studies considering these factors may provide a more nuanced understanding of nursing workforce attrition. Finally, it should be noted that since the data covers a 10-year period, some variables, such as income, may vary over the years, which may impact the model’s accuracy. Future studies should be conducted on a wider range of hospitals and a larger number of nursing personnel to address these limitations. Overall, while this study has established the foundation for applying machine learning techniques in predicting nursing turnover, further research is necessary to fully explore its potential and address areas for improvement.

## 5. Conclusions

This study developed a machine learning-based prediction model for nurse turnover in Korea, and the model showed high prediction rates. Application of the model can help improve efficiency and lower the cost of managing nursing personnel. Furthermore, the study provides a basis for applying machine learning technology in nursing human resource management.

## Figures and Tables

**Table 1:** Classification model index.

		True Class		Performance Metrics
		Positive (1)	Negative (0)	
Predicted class	Positive (1)	TP (True positive)	FP (False positive)	Accuracy: ((TP + TN)/(TP + FN + FP + TN)) Precision: (TP/(TP + FP)) Recall/Sensitivity: (TP/(TP + FN)) F1-score: (2× (Precision×Recall)/(Precision + Recall))
	Negative (0)	FN (False negative)	TN (True negative)	

FN; Wrong prediction of non-N as N, FP; wrong prediction of non-P as P, TN; N is predicted as N, TP; P is predicted as P.

**Table 2:** Sociodemographic characteristics of the employees and resignees.

Variables	Categories	Resignees (*N* = 629)		Employees (*N* = 777)	
		*N* (%)	Mean ± SD	*N* (%)	Mean ± SD
Age (yr)	20s	250 (39.7)	33.06 ± 6.13	475 (61.1)	30.95 ± 7.37
	30s	305 (48.5)		192 (24.7)	
	40s	67 (10.7)		97 (12.5)	
	50s	5 (0.8)		13 (1.7)	
	60s	2 (0.3)		0 (0.0)	
Sex	Female	577 (91.7)		718 (92.4)	
	Male	52 (8.3)		59 (7.6)	
Team	Outpatient	186 (29.6)		164 (21.1)	
	ICU	163 (25.9)		213 (27.4)	
	Ward	299 (47.5)		399 (51.4)	
	Nursing department	1 (0.2)		1 (0.1)	
Grade	Nurse	614 (97.6)		712 (91.6)	
	Nurse practitioner	15 (2.4)		65 (8.4)	
Income	Unit: 10 million won		4.19 ± 0.83		5.11 ± 0.93
Dormitory	Resident	442 (70.3)		151 (19.4)	
	Non-resident	187 (29.7)		626 (80.6)	
Married	Yes	116 (18.4)		161 (20.7)	
	No	513 (81.6)		616 (79.3)	
Distance from home to workplace	Near	542 (86.2)		715 (92.0)	
	Far	87 (13.8)		62 (8.0)	

## Data Availability

The data used to support the findings of this study are under ethical restrictions and cannot be made publicly available. Data are available from the corresponding author for researchers who meet the criteria for access to confidential data.
